# The production of alpha/beta and gamma/delta double negative (DN) T-cells and their role in the maintenance of pregnancy

**DOI:** 10.1186/s12958-015-0073-5

**Published:** 2015-07-12

**Authors:** John C. Chapman, Fae M. Chapman, Sandra D. Michael

**Affiliations:** Department of Biological Sciences, Binghamton University, Binghamton, New York 13902-6000 USA

**Keywords:** Mast cells, Sex steroids, DN pathway, DN T-cells

## Abstract

The ability of the thymus gland to convert bone marrow-derived progenitor cells into single positive (SP) T-cells is well known. In this review we present evidence that the thymus, in addition to producing SP T-cells, also has a pathway for the production of double negative (DN) T-cells. The existence of this pathway was noted during our examination of relevant literature to determine the cause of sex steroid-induced thymocyte loss. In conducting this search our objective was to answer the question of whether thymocyte loss is the end product of a typical interaction between the reproductive and immune systems, or evidence that the two systems are incompatible. We can now report that “thymocyte loss” is a normal process that occurs during the production of DN T-cells. The DN T-cell pathway is unique in that it is mediated by thymic mast cells, and becomes functional following puberty. Sex steroids initiate the development of the pathway by binding to an estrogen receptor alpha located in the outer membrane of the mast cells, causing their activation. This results in their uptake of extracellular calcium, and the production and subsequent release of histamine and serotonin. Lymphatic vessels, located in the subcapsular region of the thymus, respond to the two vasodilators by undergoing a substantial and preferential uptake of gamma/delta and alpha/beta DN T- cells. These T- cells exit the thymus via efferent lymphatic vessels and enter the lymphatic system.

The DN pathway is responsible for the production of three subsets of gamma/delta DN T-cells and one subset of alpha/beta DN T-cells. In postpubertal animals approximately 35 % of total thymocytes exit the thymus as DN T-cells, regardless of sex. In pregnant females, their levels undergo a dramatic increase. Gamma/delta DN T-cells produce cytokines that are essential for the maintenance of pregnancy.

## Background

Steroids play a commanding role in all aspects of reproduction [1]. They do this through the mediation of steroid receptors, a process that is purported to involve components of the immune system [2, 3]. However, research conducted during the development of oral contraceptives suggests that a ligand-receptor interaction between the two systems may not be possible. This became apparent when it was found that injecting female rats with estrogen and testosterone caused the thymus to suffer a severe loss of thymocytes and to undergo thymic involution [4]. Although this finding was regarded as atypical and due to exposing the thymus to excessive levels of the two steroids [4], a more recent report found that physiological levels of estrogen also cause thymocyte loss and thymic involution [5]. Taken *in toto*, these studies have led to the theory that sex steroids initiate, and then perpetuate the aging process of the immune system [6]. This would suggest that the two systems are ill-suited for each other. We disagree with this premise and will present evidence to show that thymocyte loss, instead of being due to incompatibility, results from a sex steroid-induced release of γδ and αβ double-negative [DN] T- cells into the lymphatic system. In brief, the discharge of these T-cells occurs when sex steroids bind to the estrogen receptor alpha [7] of thymic mast cells. Mast cell activation, coincident with a rapid influx of extracellular calcium, results in the release of vasodilators such as histamine and serotonin [8]. Nearby lymphatic vessels become enlarged and undergo a preferential and significant uptake of the aforementioned DN T- cells. The T-cells then exit the thymus via efferent lymphatic vessels and enter the lymphatic system. These DN T-cells play a key role in the maintenance of pregnancy.

## Review

After exposure to hydrocortisone and dexamethasone, thymocytes become apoptotic and undergo cell death [9, 10]. Whether or not sex steroids cause thymocyte loss by apoptosis was examined in a number of studies in which animals were subjected to estrogen administration. Unfortunately, the results were notable for their lack of consensus. Estrogen treatment in some studies resulted in an increase in the rate of thymocyte apoptosis [11–13], whereas in other reports, estrogen treatment produced little or no evidence of apoptotic death [14, 15]. In a further study of the phenonomen, Zoller et al. [5] found that pregnant mice undergo extensive thymocyte loss and thymic involution without thymocyte apoptosis ever taking place. In pregnant mice, the levels of estrogen range between 7 ng/ml to 13 ng/ml [16]. Studies that reported a high incidence of thymocyte apoptosis injected the animals with levels of estrogen far in excess of these values [11–13]. Thus, without evidence to show that physiological levels of estrogen cause apoptosis, this process can be ruled out as the reason for thymic involution and thymocyte loss.

Some investigators have proposed that thymocyte loss takes place because estrogen blocks T-cell production at the precursor level. This premise came from a study in which estrogen treatment resulted in an increase in the levels of the earliest CD44^+^ progenitors and a depletion of all defined thymocyte subsets of CD4^+^ and CD8^+^ T-cells [17]. Other researchers have proposed that thymic involution is due to an estrogen-induced reduction in early thymic progenitors [15]. These studies suggest the possibility that thymocyte loss is the result of an alteration in T-cell production.

Martin et al. [18, 19], using light and electron microscopy, observed an estrogen-induced loss of thymocytes in the subcapsular and deep cortex of the rat thymus. In the medullary region, they found evidence of an increase in the vascular permeability of blood vessels located near the corticomedullary junction. Lymphocytes were often seen migrating through the enlarged walls of these blood vessels. They concluded that “the release of lymphocytes from the thymus seems to be the main factor inducing thymic involution.” Others have observed that the lymphatic vessels in involuted thymuses are packed with lymphocytes (T-cells) [20, 21].

Although not identified as such, these lymphatic vessels would have to be efferent lymphatic vessels, since the thymus lacks the afferent variety [22, 23], an important distinction.

Oner and Ozan [24] reported that prolonged treatment of female rats with either testosterone or estrogen (daily for 3 weeks) caused extensive thymic involution. This involution was accompanied by a loss of thymocytes in the subcapsular region as well as in the deep cortex. Blood vessels in the thymic medulla were also enlarged, as was noted in the report by Martin et al. [18, 19]. The most significant finding by Oner and Ozan, however, was the identification of mast cells in connective tissue of the thymic capsule and in the stroma of the thymic medulla. In untreated control rats, mast cells were sparsely distributed, whereas in steroid-treated animals, they were increased in number and often found in clumps. The fact that mast cells secrete vasodilators leaves little doubt as to the cause of the increase in vascular permeability; which may be the reason why involuted thymuses were packed with lymphocytes [20, 21]. As to the identity of these lymphocytes, studies of estrogen-injected [25, 26] and thymic-implanted nude mice [27] revealed that “thymocyte loss” was the result of the discharge of two subsets of DN T-cells [25, 26]. One subset had a typical αβ T-cell receptor (TCR), and the other had a unique γδ TCR.

### T-cell production

The thymus gland consists of two distinct lobes, each composed of a central medulla and an outer cortex. Two layers of connective tissue, separated by a sinus, encapsulate both lobes. In most species, the capsule gives rise to trabeculae that penetrate the cortex and terminate at the corticomedullary junction, thereby providing a structural link to the medulla. A basement membrane supports a specialized flattened epithelium lining the subcapsule and trabeculae. Arteries travel within the capsule and then either enter the cortex as arterioles or continue within the trabeculae until they reach the corticomedullary junction, where they pass into the medulla. Arterioles become progressively smaller and continue throughout the thymus as capillaries, undergoing eventual transformation into venous capillaries and subsequent enlargement to form postcapillary venules (PCVs). These venules ultimately lead to major blood vessels that travel back to the trabeculae, where they leave in close proximity to the incoming arteries [21, 22, 24, 28–30].

The distribution of blood and lymphatic vessels (LVs) is not uniform. For example, the cortex lacks PCVs, whereas the medulla contains a large number [21, 22]. In addition, the cortex contains a small contingent of branched LVs, located mainly in the subcapsular region [31]. These vessels extend into the capsule and extralobular region and connect to efferent lymphatic vessels (ELVs) [21, 22, 31]. In the medulla, LVs are more plentiful and are localized in the region of the corticomedullary junction. These connect with ELVs in the trabeculae. Mast cells are absent from the cortex, but are found nearby in the connective tissue of the capsule [24]. In the medulla, mast cells are located in proximity to both LVs and PCVs [24, 32, 33]. Notably, in the involuted thymus the number of mast cells is significantly increased [24, 34, 35].

T-cell progenitors produced in the bone marrow reach the thymus via the arterial branch of the circulatory system. Upon entering the gland, they travel through arterioles as well as arterial and venous capillaries until they arrive at the PCVs. The progenitors then pass into the thymic stroma, using a process referred to as extravasation, or diapedesis. Diapedesis takes place in vessels that have walls of endothelium and lack a muscular layer [36], such as PCVs and LVs. Endothelial cells are unique in that lymphocytes are able to insinuate themselves between cellular junctions, then pass either into, or out of, the thymic stroma [37–39]. Lymphocyte movement is aided by estrogen-activated mast cells through their production of histamine and serotonin, which in turn, causes a widening of the cellular junctions of the endothelial cells [36]. Diapedesis in PCVs is unidirectional and limited to lymphocyte movement from the lumen into the thymus [40, 41]. For passage out of the thymus, the T-cells utilize LVs [42–46], since these are capable of reverse diapedesis [47, 48].

**Fig. 1:**
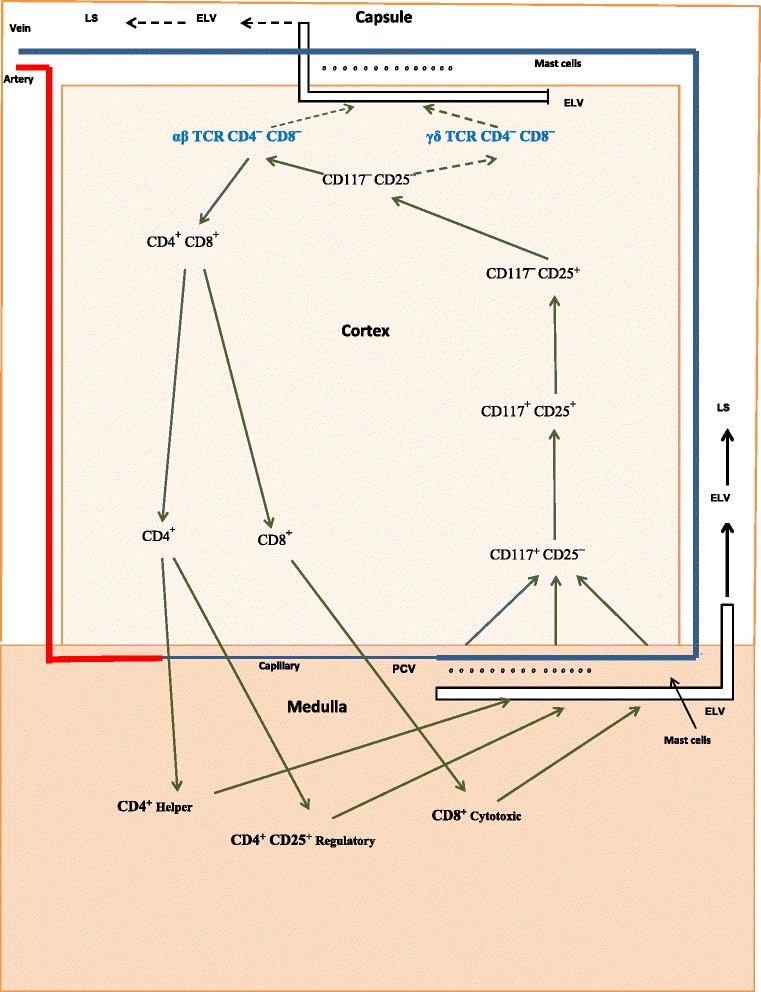
Proposed pathways for the production of T-cells in prepubertal mice. Progenitor cells enter the thymus via postcapillary venules (PCVs) located in the medulla and as T-cells leave by way of efferent lymphatic vessels (ELVs) located in the subcapsular cortex and in the medulla. In prepubertal mice, the majority of thymocytes traverse the classic developmental pathway and as SP T-cells enter the lymphatic system (LS) (*solid black arrows*) via ELVs located in the medulla. Lesser numbers of thymocytes enter the LS (*dashed black arrows*) as DN T-cells through ELVs located in the subscapular region

**Fig. 2:**
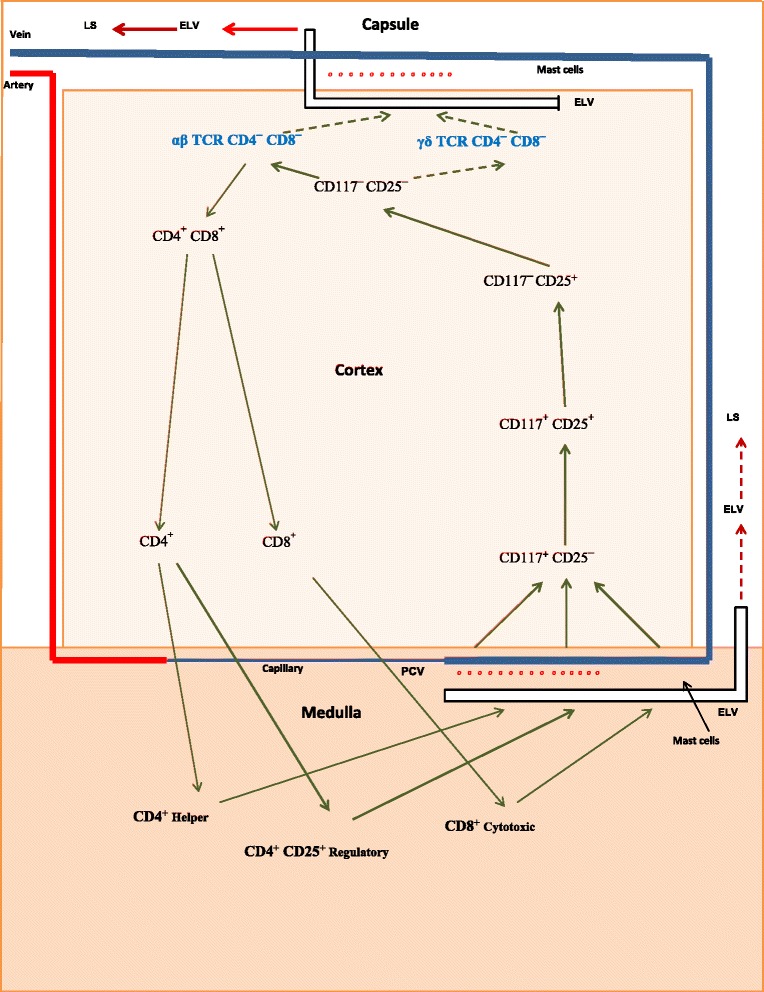
Proposed pathways for the production of T-cells in postpubertal mice. Progenitor cells enter the thymus via postcapillary venules (PCVs) located in the medulla and as T-cells leave by way of efferent lymphatic vessels (ELVs) located in the subcapsular cortex and in the medulla. In postpubertal mice, mast cell activation (*red dots*) results in large numbers of thymocytes exiting the classic pathway as DN T-cells and entering the LS (*solid red arrows*) via ELVs located in the subcapsular region. Lesser numbers of thymocytes remain in the classic pathway and enter the LS (*dashed red arrows*) as SP T-cells via ELVs located in the medulla

Figures 1 and 2 are graphic representations of thymocyte development in pre- and postpubertal mice. Shown in each figure are four spatially defined developmental stages in the cortex that Lind and colleagues [49] have mapped using the progenitor markers, CD117 and CD25. Differential expression of these two markers reflects developmental changes in the thymocytes as they move outward from the corticomedullary junction into the cortex. In this process, thymocyte movement is aided in large part by an interaction between chemokines produced by cortical epithelial cells in specific areas of the cortex, and thymocyte chemokine receptors [50]. Stage 1 (CD117^+^CD25^—^) begins at the corticomedullary junction and is characterized by thymocytes with multilineage potential. These cells, in addition to giving rise to T lymphocytes, can also evolve into B lymphocytes, as well as dendritic and NK cells. Cells that reach stage 2 (CD117^+^CD25^+^) no longer have the ability to become B lymphocytes and NK cells, but can give rise to αβ T-cells, γδ T-cells, and dendritic cells. Intracellular CD3ε protein is detected at this stage [51]. In addition, a significant amount of thymocyte proliferation occurs at stage 2. Cells that reach stage 3 (CD117^—^CD25^+^) are committed to T-cell lineage. Intracellular CD3ε protein synthesis continues unabated. TCR β protein is first detected at this stage. Cells that express productive rearrangements of TCR β with an α chain are selected to proliferate and proceed to stage 4, a process termed as β selection. At stage 4 (CD117^—^CD25^—^), thymocytes have reached the subcapsular region of the cortex with their TCR in place and γ and δ binding components added to the CD3 complex. Most have traversed the αβ TCR developmental pathway and are characterized as αβ CD4^—^CD8^—^ double-negative (DN) T-cells. Thymocytes that have developed a γδ TCR are referred to as γδ DN T-cells. Their numbers comprise 5-10 % of total DN T-cells [27, 52].

### DN T-cell pathway

Gamma/delta T-cells are not found in the thymus beyond stage 4 of development [51]. This suggests: 1) an absence of thymic tissue specifically dedicated to the continuation of their chemokine-facilitated travel; and 2) a strong probability that they leave the thymus directly after they are produced. Lymphatic vessels located nearby in the subcapsular cortex are very likely their means of exit. In mice, the DN pathway is operational shortly shortly after birth, with DN T-cells being found in the liver and spleen of 4-day-old animals [52, 53]. Notably, the levels of αβ DN T-cells exceed that of γδ DN T-cells by a factor of 4:1. Shown in Fig. 1 are the proposed exit pathways of γδ DN T-cells and αβ DN T-cells in prepubertal mice. As is indicated, most T-cells leave the thymus via ELVs located in the medulla (solid black arrows). However, in postpubertal mice (Fig. 2) a large number of γδ DN T-cells and αβ DN T-cells exit the thymus via ELVs located in the subcapsular cortex (solid red arrows) as the result of a sex steroid-induced activation of thymic mast cells.

Estrogen activation of mast cells takes place via a membrane-associated (non-genomic) estrogen receptor-α (ER-α) [7]. This activation results in an influx of extra-cellular calcium and the synthesis and release of granules of histamine and serotonin [8]. Mast cell activation can be achieved with concentrations of estrogen between 10^−11^ M and 10^−9^ M (2.7 pg/ml to 270 pg/ml) [54]. Testosterone activation requires levels that are 10 times that of estrogen [55]. Activation by the weak androgen, dehydroepiandrosterone (DHEA), necessitates levels that are 1000 times that of estrogen [56, 57]. Dihydrotestosterone (DHT) is also a mast cell activator [58]. Progesterone is an inhibitor of estrogen activation [59].

In postpubertal animals, endogenous sex steroids attain levels that are fully capable of activating thymic mast cells. For example, circulating levels of testosterone in male mice and rats average 18.7 ng/ml and 5.8 ng/ml, respectively [60]. In nonpregnant female mice and rats, the levels of estrogen are 66 pg/ml and 30.6 pg/ml, respectively [61, 62], and in pregnant mice, estrogen levels range from 7 ng/ml to 13 ng/ml [16]. Strong evidence that the ER-α plays a role in estrogen-induced thymic involution is indicated by studies of estrogen receptor knockout mice (ERKO). In these animals, the ER-α is nonfunctional; consequently, the thymus undergoes only minimal estrogen-induced involution [63, 64].

### Classic T-cell pathway

In contrast to the fate of γδ DN T-cells, αβ DN T cells retain the option of continuing their development in the thymus. This choice is exercised when CD4 and CD8 markers are expressed, and αβ DN T-cells become CD4^+^ CD8^+^ double-positive (DP) T-cells. In utilizing this option, DP T-cells apparently lose the ability to access the DN pathway. This is either because they are restricted from doing so or have left the area of the subcapsular LVs. Abo’s group reports a total absence of DP T-cells in the pool of SP T-cells and DN T-cells found in the liver of estrogen-injected mice [25]. In the next developmental stage, DP T-cells undergo positive selection, a procedure concurrent with the production of two subsets of single-positive (SP) MHC restricted T-cells. These subsets are CD4^+^ (class II MHC-restricted) and CD8^+^ (class I-MHC restricted) T-cells, and as such, they continue on into the medulla. Here they undergo negative deletion, a process in which their αβ TCRs are exposed to ectopic self-antigens. Production of these antigens is under the direction of the autoimmune regulator (Aire) promotor [65]. Fully mature CD4^+^_Helper_, CD4^+^ CD25^+^ Foxp3^+^_Regulatory_, and CD8^+^_Cytotoxic_ T-cells exit the thymus via LVs located in the medulla (Fig. 1, solid black arrows; Fig. 2, dashed red arrows).

### Interaction between DN and SP pathways

It should be noted that the permeability of all LVs and PCVs is increased through the combined action of sex steroids and mast cells. This results in an increased entry of T-cell progenitors and an enhancement in the exit rate of DN T-cells. To gain an appreciation of the levels of thymocytes that exit the thymus via the DN pathway, one only has to measure the total number of thymocytes prior to, and after castration. Fortunately, this has been done by a number of researchers. For example, Pesic et al. [66] reported that thymocyte levels in castrate and intact 60-day-old Albino-Oxford male rats were 1050 × 10^6^ and 650 × 10^6^, respectively. This would suggest that mast cell activation has facilitated the exit of 38 % of total thymocytes. Notably, these thymocytes were reported to originate from the cortex. In a study of intact and castrated 60-day-old female Sprague–Dawley rats [57], the results indicated that estrogen caused 44 % of total thymocytes to exit via the DN pathway. Findings from a third study of male and female adult Wistar-albino rats [24] revealed that testosterone and estrogen affected a reduction of 31 % and 30 % of total thymocytes, respectively. These studies demonstrate the effect of sex steroids in altering the dynamics of T-cell production. In the castrate animal, the thymus produces mainly SP T-cells. Their production time takes 3–5 days in the cortex and 12–16 days in the medulla [67], for a total of ~21 days. In the intact animal, a significant number of DN T-cells exit the thymus via the DN pathway. Their total production time is 3–5 days. In these animals, the reports of a reduction in thymocyte levels of ~ 35 % [24, 57, 66] strongly indicates that progenitor replacement does not keep pace with DN T-cell production.

**Fig. 3:**
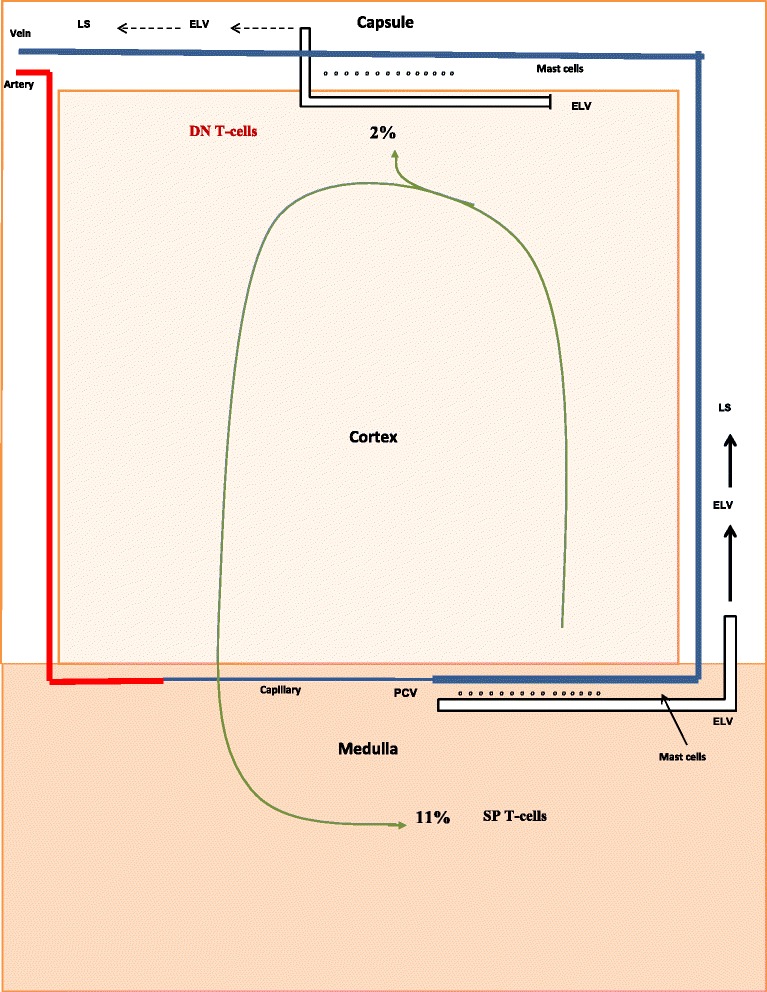
Production of DN T-cells and SP T-cells by castrate adult animals. Shown are the percentages of DN T-cells and SP T-cells produced by castrate adult animals. The numerical values were determined from the data of Pesic et al. [66]

**Fig. 4:**
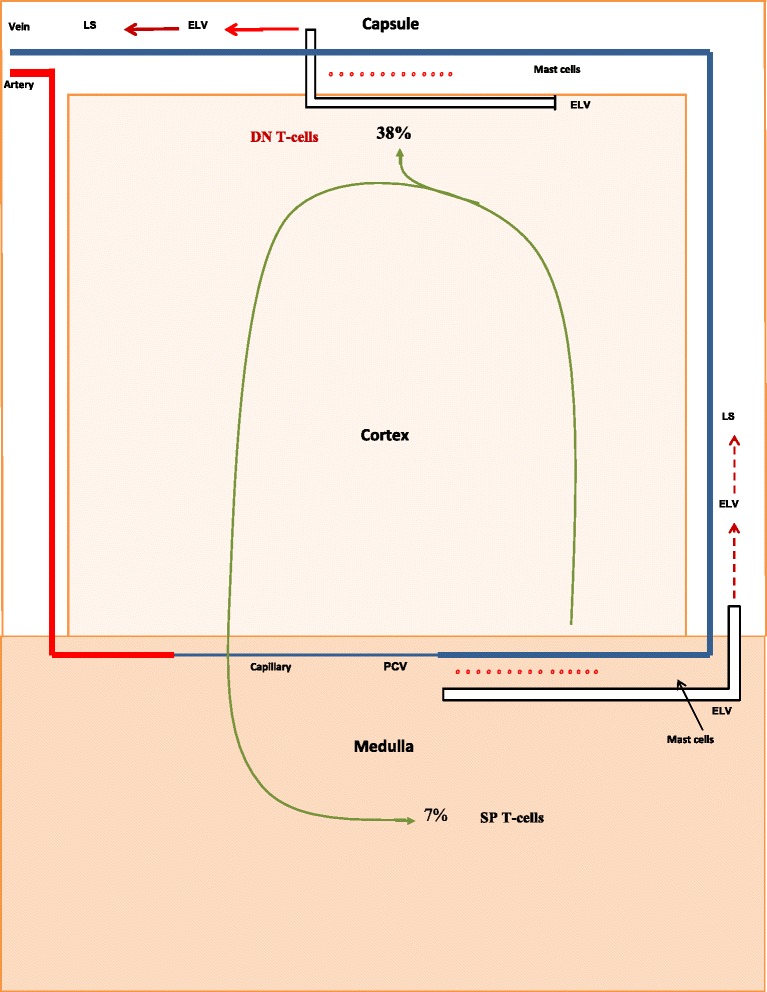
Production of DN T-cells and SP T-cells by intact adult animals. Shown are the percentages of DN T-cells and SP T-cells produced by intact adult animals. The numerical values were determined from the data of Pesic et al. [66]

Pesic et al. [66] also measured thymocyte levels in the cortex and medulla of intact and castrate male rats. With this information we were able to examine the effect of the discharge of DN T-cells in altering the levels of SP T-cells. For example, in the castrate animal (Fig. 3) a comparison between thymocyte levels in the cortex and medulla indicates that 2 % of total thymocytes leave via the DN pathway, and 11 % reach the medulla to become SP T-cells. Without castration (Fig. 4), a similar comparison suggests that 38 % of total thymocytes exit via the DN pathway and only 7 % reach the medulla. Thus, the production of DN T-cells is the result of a proverbial “fork in the road” of T-cell development. Thymocytes can either leave the thymus as DN T-cells, or they can remain in the classic T-cell pathway and exit as SP T-cells. Their pathway of development is determined by sex steroids. For example, during pregnancy when estrogen levels are at their highest, large numbers of T-cells utilize the DN pathway. As a consequence, the production of SP T-cells is at its nadir [15]. We estimate that during pregnancy only 2 % of total thymocytes reach the medulla.

### DN T-cells

DN T-cells do not undergo positive selection (Figs. 1 and 2). Consequently, they lack MHC restriction. This factor, in combination with their unique TCR, produces binding characteristics for γδ DN T-cells that differ substantially from that of MHC restricted αβ T-cells. Where the latter bind to fragments (epitopes) of foreign antigen held within the cleft of a class I or class II MHC molecule [68], γδ DN T-cells do not. Instead, their binding to foreign antigen is based on the conformational shape of the intact antigen, similar to that of antibodies, and independent of MHC involvement [69].

There are three major subsets of γδ DN T-cells, one of which is cytolytic. In humans this subset has been characterized via its TCR as a Vγ9Vδ2 T-cell [69, 70]. When activated, they secrete interleukin-2 (IL-2), interferon-γ (IFN-γ), and tumor necrosis factor-β (TNF-β) [71]. These cytokines promote inflammation, cytotoxicity, and delayed-type hypersensitivity (DTH) [72]. Vγ9Vδ2 T-cells are unconventional in that non-proteins such as isoprenoids and alkylamines cause their activation [69]. Their venue of immunological activity is in the peripheral bloodstream [70, 71]. Here they have an important role in both tumor cell surveillance and anti-infective immunity [73]. The second subset of γδ DN T-cells has all the characteristics of the Vγ9Vδ2 T-cells, except they are not cytolytic. The reason they are not is because they have an intermediate and incompletely expressed TCR/CD3 binding complex [74–76]. Henceforth they will be referred to as γδ DN (int TCR/CD3) T-cells. Rather than being in the bloodstream, these T-cells reside in the intraepithelial lymphocyte compartments of specific tissues such as skin, intestine, respiratory tract and uterus [69, 74]. The third subset of γδ DN T-cells are regulatory. In mice they are characterized as Vγ6Vδ1 regulatory T-cells [77]. Activation of these γδ DN regulatory T-cells results in the production of IL-10 and transforming growth factor-β (TGF-β) [76, 78]. These cytokines control the action of cytotoxic T cells, NK cells, macrophages, dendritic cells and B cells [79]. The γδ DN regulatory T-cells are also restricted to the intraepithelial lymphocyte compartments of specific tissues [79]. In the uterus they play a significant role in the maintenance of pregnancy.

Alpha/beta DN T-cells are cytolytic [80] and produce IL-4, IFN-γ, and TNF-β, but not IL-2 [81]. These T-cells have a significant role in the control of intracellular bacterial infection [82].

### Immunomodulation, DN T-cells, and the maintenance of pregnancy

The maintenance of pregnancy depends, to a large extent, on the avoidance of maternal rejection. This is dealt with through the construction of an immunological barrier using cells that lack the ability to express classical HLA-A and HLA-B products [83]. This produces a protective cocoon (trophoblast) in which MHC class I and MHC class II molecules are either missing or non-functional [84]; as a consequence, the processing and presentation of antigens by MHC molecules cannot take place. SP T-cells are thus eliminated as a rejection factor, leaving only γδ DN T-cells to respond to the trophoblast. Instead of rejection, however, these T-cells are essential for the maintenance of pregnancy. The complexity of their overall role and the need for coordination requires extensive communication between γδ DN T-cells and the decidua and trophoblast. The trophoblast, for example, initiates contact with a variety of immune cells through its production and release of chemokines. These are small proteins that act as ligands to immune cell receptors. The binding of these unique ligands to specific receptors results in the production of adhesion molecules by respondent cells, thereby giving them the means to adhere to the endothelium of blood vessels. With this ability they are able to follow a chemokine concentration gradient to its source [85]. Cytokines produced by γδ DN T-cells, in contrast, encompass a broader application than chemokines in that they influence the growth and receptivity of specific cell populations.

The trophoblast attracts immune cells to the fetal-maternal interface through its production of the chemokines CXCL12 and CXCL16. For example, CXCL12 recruits NK cells that have CXCR3 and CXCR4 receptors [86, 87], and CXCL16 recruits αβ T-cells, γδ DN T-cells, and monocytes through its interaction with CXCR6 receptors [88]. Analyses of the decidua during early to mid-pregnancy has identified the presence of the following cells: 1) γδ DN regulatory T-cells; 2) γδ DN (int TCR/CD3) T-cells; 3) CD8^+^ cytotoxic T-cells; 4) CD4^+^ CD25^+^ Foxp3^+^ regulatory T-cells; 5) NK cells; 6) dendritic cells; 7) macrophages; and 8) neutrophils [75, 89, 90]. These cells have all reached the decidua via the cardiovascular system, with two exceptions. The exceptions are γδ DN regulatory T-cells and γδ DN (int TCR/CD3) T-cells. These two subsets are part of a group that obtains access to their target tissues via the lymphatic system [69, 74, 75, 78, 91].

In nonpregnant women, mice, rats, and rabbits, the lymphatic system does not extend beyond the myometrium [92–94]. Therefore, during early pregnancy, γδ DN regulatory T-cells and γδ DN (int TCR/CD3) T-cells are unable to respond to CXCL16 until lymphangiogenesis (lymphatic vessel growth) has linked the endometrium to the lymphatic system. As a consequence, these T-cells are the last to reach the fetal-maternal interface. Their late arrival indicates the likelihood that lymphangiogenesis does not require their input, at least at this point. Their participation in the process comes later and is essential for the maintenance of pregnancy.

Gamma/delta DN cytolytic T-cells are found in the uterus during the early stages of pregnancy [95]. Their presence in this location is very likely due to CXCL16. However, the main function of these T-cells is to detect and destroy bacteria, and they are highly cytolytic. Thus, it is unusual for these cells to be in close proximity to the trophoblast without causing its destruction [96]. To protect the trophoblast could be the reason why a large number of CD4^+^ CD25^+^ Foxp3^+^ regulatory T-cells reside in the decidua [89]. These regulatory T-cells are fully capable of eliminating γδ DN cytolytic T-cells [97, 98]. It is noteworthy that Foxp3^+^ regulatory T-cells are among the first immune cells to enter the uterus, indicating that they are in place prior to the entry of the γδ DN cytolytic T-cells [99]. Levels of Foxp3^+^ regulatory T-cells undergo a significant increase during pregnancy [5, 89, 100, 101], with the decidua being the major recipient of their enhanced production [89]. It should be noted that this form of protection for the trophoblast has an upper limit since excess numbers of peripheral γδ DN cytolytic T-cells can cause abortion [96, 102, 103]. It was not reported in these studies if the increase in γδ DN cytolytic T-cells was due to acute bacterial infection [104, 105]. Putative evidence of the involvement of Foxp3^+^ regulatory T-cells in preventing abortion is indicated by reports that women with decreased levels of these T-cells suffer from recurrent miscarriages [106–108].

NK cells play a significant role in the creation of blood and lymphatic vessels. Their major responsibility is to produce a large number of cytokines. These include vascular endothelial growth factor (VEGF), fibroblast growth factor (FGF), TNF-β, IFN-γ, and the angiopoietins, to name a few [109]. Blood-borne NK cells are cytolytic and fully capable of destroying the trophoblast [110]. However, unlike the γδ DN cytolytic T-cells, they are not eliminated. Instead, they are converted into noncytolytic NK cells. This transformation is under the control of TGF-β, and involves converting cytolytic CD56^dim^ CD16^+^ peripheral NK cells (CD16^+^ pNK cells) into noncytolytic CD56^bright^ CD16^—^ uterine NK cells (CD16^—^ uNK cells) [111–113]. The initial source of TGF-β for pNK cell conversion is provided by the male, and TGF-β reaches the decidual area via the ejaculate [114–116]. TGF-β is also produced by decidual stromal cells [112]. However, the overall supply of TGF-β is not inexhaustible. The TGF-β derived from the ejaculate is limited for obvious reasons, and the ability of stromal cells to produce the cytokine is seriously compromised. This is because TGF-β is involved in two simultaneous and conflicting operations. In addition to converting pNK cells into uNK cells, TGF-β is also involved in implantation. Its role in this process is to initiate the apoptotic destruction of decidual stromal cells.

Shooner et al. [117] noted that stromal cells of the pregnant rat uterus undergo a TGF-β-induced increase in apoptosis between day 5 and day 14 of pregnancy. During this period, the loss in stromal cells is correlated with decreased production of the two isoforms, TGF-β1 and TGF-β2. After day 14, only limited quantities of TGF-β are produced by stromal cell survivors. Without replenishment, the decrease in TGF-β could have a serious impact on the transformation of pNK cells to uNK cells. Red-Horse [94] noted that lymphatic vessels in the endometrial area of pregnant mice begin their development between embryonic day 9.0 and day 9.5. This would indicate that these lymphatic vessels have ~ 5 days to complete their development before TGF-β is seriously depleted. This timeframe is critical since γδ DN regulatory T-cells, a major source of TGF-β, can only reach the fetal-maternal interface via the newly-formed lymphatic vessels.

TGF-β is regarded as a pleiotropic cytokine. This characteristic is obvious during the maintenance of pregnancy. Here, the cytokine has a significant impact on lymphangiogenesis by controlling levels of pNK cells [112]. However, while TGF-α is performing this function it is undergoing self-destruction by initiating the apoptosis of decidual stromal cells [117]. Both processes are essential for the maintenance of pregnancy. The prospect of the cytokine being depleted during implantation is troublesome. One could visualize scenarios in which the levels of stromal cells were lower than normal, or where TGF-β-induced stromal cell apoptosis occurred at a faster rate. In these instances, implantation would be successful, whereas a scarcity of TGF-β could alter the formation of lymphatic vessels. If this occurred, it would prevent γδ DN regulatory T-cells from reaching the fetal-maternal interface. The loss of a major source of TGF-β could impede the conversion of pNK cells to uNK cells. Notably, a number of studies have reported that excess levels of pNK in pregnant women are highly correlated with recurrent spontaneous abortion [118–125].

## Conclusions

In this review we have presented evidence indicating that sex steroids initiate a pathway for the production of DN T-cells. This is done through the mediation of thymic mast cells. In females, the DN pathway is controlled by estrogen. During pregnancy, estrogen levels increase [126], causing the production of DN T-cells to take priority over the production of SP T-cells. This guarantees that the trophoblast will have a plentiful and ever increasing supply of γδ DN regulatory T-cells and γδ DN (int TCR/CD3) T-cells. In addition to their increased production, these two T-cell subsets are specifically guided to the trophoblast by CXCL16; and, after travelling through efferent lymphatic vessels and newly-formed endometrial lymphatic vessels, they perform their role in the maintenance of pregnancy. This role involves the production of cytokines; a process that requires T-cell activation. Since γδ DN T-cells and the trophoblast both lack MHC restriction, this activation occurs by binding to an intact antigen. Heyborne et al. have proposed that heat shock protein-60 (HSP-60) has all the attributes to be that antigen [127].

Activation of γδ DN (int TCR/CD3) T-cells results in the production of TNF-α and IFN-γ. These cytokines are responsible for maintaining the integrity of the blood and lymphatic vessels [128–130]. As important as this is to maintaining pregnancy, activation of γδ DN regulatory T-cells, and their production of IL-10 and TGF-β, is of far greater consequence. IL-10 by itself promotes trophoblast invasion, and suppresses trophoblast apoptosis [78]; whereas TGF-β is involved in lymphangiogenesis and implantation [112, 117]. However, when IL-10 and TGF-β act in combination, the two cytokines perform a synergistic suppression of the cytolytic activities of γδ DN cytolytic T-cells and pNK cells [131]. This would strongly suggest that the two cytokines are supplanting, or adding to, the role of the CD4^+^ CD25^+^ Foxp3^+^ regulatory T-cells in protecting the trophoblast. The importance of γδ DN regulatory T-cells in the maintenance of pregnancy has been demonstrated by Arck et al. [96]. This group found that the rate of abortion in pregnant mice underwent a significant increase when the animals were given monoclonal antibodies against γδ DN regulatory T-cells. Notably, these mice were injected with the antibodies after 8.5 days of gestation, coincident with the time when γδ DN regulatory T-cells first reach the decidua, and 3.5 days after implantation. One could speculate that construction of endometrial lymphatic vessels was the reason that γδ DN regulatory T-cells did not reach the decidua prior to 8.5 days of gestation.

The production of DN T-cells in anti-infective immunity can take place without sex steroid involvment. For example, in response to the direct invasion of bacteria and tumor cells [132–134], mast cell activation occurs when the Fc component of either IgG or IgE antibodies bind to FcγR and FcεR receptors. The two receptors are also located on the membrane of mast cells, and the immunoglobulins are the product of an antigen-induced activation of B cells and their subsequent differentiation into antibody-secreting plasma cells. With this brief description of the antibody-induced initiation of the DN T-cell pathway we conclude this review. We should point out that this is only one of the ways that γδ DN T-cells act as early sensors of stress and infection. Admittedly, much of this review has been about the role of γδ DN T-cells in reproduction. However, in doing this we have presented a careful overview of current research on sex steroid-induced “thymocyte loss.” We look forward to future research to advance our understanding of the role of γδ DN T-cells in reproduction as well as in immunology.

## Abbreviations

ELVs: Efferent lymphatic vessels
LVs: Lymphatic vessels
PCVs: Postcapillary venules
DN T-cells: CD4^—^ CD8^—^ T-cells
SP T-cells: CD4^+^ CD8^—^ T-cells and CD4^—^CD8^+^ T-cells

## References

[1] Albrecht ED, Aberdeen GW, Pepe GJ (2000). The role of estrogen in the maintenance of primate pregnancy. Am J Obstet Gynecol.

[2] Olsen NJ, Kovacs WJ (1996). Gonadal steroids and immunity. Endocr Rev.

[3] Olsen NJ, Olson G, Viselli SM, Gu X, Kovacs WJ (2001). Androgen receptors in thymic epithelium modulate thymus size and thymocyte development. Endocrinology.

[4] Kuhl H, Gross M, Schneider M, Weber W, Mehlis W, Stegmuller M, Taubert HD (1983). The effect of sex steroids and hormonal contraceptives upon thymus and spleen on intact female rats. Contraception.

[5] Zoller AL, Schnell FJ, Kersh GJ (2007). Murine pregnancy leads to reduced proliferation of maternal thymocytes and decreased thymic emigration. Immunology.

[6] Hince M, Sakkal S, Vlahos K, Dudakov J, Boyd R, Chidgey A (2008). The role of sex steroids and gonadectomy in the control of thymic involution. Cell Immunol.

[7] Zaitsu M, Narita S, Lambert KC, Grady JJ, Estes DM, Curran EM, Brooks EG, Watson CS, Goldblum RM, Midoro-Horiuti T (2007). Estradiol activates mast cells via a non- genomic estrogen receptor-α and calcium influx. Mol Immunol.

[8] Metcalfe DD (2008). Mast cells and mastocytosis. Blood.

[9] Maruyama S, Tsukahara A, Suzuki S, Tada T, Minagawa M, Watanabe H, Hatakeyama K, Abo T (1999). Quick recovery in the generation of self-reactive CD4^low^ natural killer (NK) T cells by an alternative pathway when restored from acute thymic atrophy. Clin Exp Immunol.

[10] Chmeilewski V, Drupt F, Morfin R (2000). Dexamethasone induced apoptosis of mouse thymocytes: prevention by native 7α-hydroxysteroids. Immunol Cell Biol.

[11] Yao G, Hou Y (2004). Thymic atrophy via estrogen-induced apoptosis is related to Fas/FasL pathway. Int Immunopharmacol.

[12] Okasha SA, Ryu S, Do Y, McKallip RJ, Nagarkatti M, Nagarkatti PS (2001). Evidence for estradiol-induced apoptosis and dysregulated T cell maturation in the thymus. Toxicology.

[13] Do Y, Ryu S, Nagarkatti M, Nagarkatti PS (2002). Role of death receptor pathway in estradiol-induced T-cell apoptosis in vivo. Toxicol Sci.

[14] Staples JE, Fiore NC, Frazier DE, Gasiewicz TA, Silverstone AE (1998). Overexpression of the anti-apoptotic oncogene, bcl-2, in the thymus does not prevent atrophy induced by estradiol or 2,3,7,8-tetrachlorodibenzo-p-dioxin. Toxicol Appl Pharmacol.

[15] Zoller AL, Kersh GJ (2006). Estrogen induces thymic atrophy by eliminating early thymic progenitors and inhibiting proliferation of β-selected thymocytes. J Immunol.

[16] Bebo BF, Fyfe-Johnson A, Adlard K, Beam AG, Vandenbark AA, Offner H (2001). Low-dose estrogen therapy ameliorates experimental autoimmune encephalomyelitis in two different inbred mouse strains. J Immunol.

[17] Rijhsinghani AG, Thompson K, Bhatia SK, Waldschmidt TJ (1996). Estrogen blocks early T cell development in the thymus. Am J Reprod Immunol.

[18] Martin A, Alonso LM, Gomez del Moral M, Zapata AG (1994). Ultrastructural changes in the adult rat thymus after estradiol benzoate treatment. Tissue Cell.

[19] Martin A, Alonso L, Gomez del Moral M, Zapata AG (1994). Morphometrical changes in the rat thymic lymphoid cells after treatment with two different doses of estradiol benzoate. Histol Histopath.

[20] Kato S (1988). Intralobular lymphatic vessels and their relationship to blood vessels in the mouse thymus. Light- and electron-microscopic study. Cell Tissue Res.

[21] Kato S (1997). Thymic microvascular system. Microsc Res Tech.

[22] Weiss L (1963). Electron microscopic, observations on the vascular barrier in the cortex of the thymus of the mouse. Anat Rec.

[23] Pearse G (2006). Normal structure, function and histology of the thymus. Toxicol Pathol.

[24] Oner H, Ozan E (2002). Effects of gonadal hormones on thymus gland after bilateral ovariectomy and orchidectomy in rats. Arch Androl.

[25] Okuyama R, Abo T, Seki S, Ohteki T, Sugiura K, Kusumi A, Kumagai K (1992). Estrogen administration activates extrathymic T cell differentiation in the liver. J Exp Med.

[26] Kimura M, Hanawa H, Watanabe H, Ogawa M, Abo T (1995). Synchronous expansion of intermediate TCR cells in the liver and uterus during pregnancy. Cell Immunol.

[27] Pardoll DM, Fowlkes BJ, Lew AM, Maloy WL, Weston MA, Bluestone JA, Schwartz RH, Coligan JE, Kruisbeek AM (1988). Thymus-dependent and thymus-independent developmental pathways for peripheral T cell receptor-γδ-bearing lymphocytes. J Immunol.

[28] Boyd RL, Tucek CL, Godfrey DI, Izon DJ, Wilson TJ, Davidson NJ, Bean AG, Ladyman HM, Ritter MA, Hugo P (1993). The thymic microenvironment. Immunol Today.

[29] Kendall MD (1991). Functional anatomy of the thymic microenvironment. J Anat.

[30] Egerton M, Scollay R, Shortman K (1990). Kinetics of mature T-cell development in the thymus. Proc Natl Acad Sci U S A.

[31] Odaka C, Morisada T, Oike Y, Suda T (2006). Distribution of lymphatic vessels in mouse thymus: immunofluorescence analysis. Cell Tissue Res.

[32] Yong LC, Watkins SG, Boland JE (1979). The mast cell: III. Distribution and maturation in various organs of the young rat. Pathology.

[33] Raica M, Cimpean AM, Nico B, Guidolin D, Ribatti D (2010). A comparative study of the spatial distribution of mast cells and microvessels in the foetal and adult thymus and thymoma. Int J Exp Pathol.

[34] Ruitenberg EJ, Buys J (1980). Thymus atrophy during early pregnancy and its effect on a Trichinella spiralis infection in mice, including intestinal pathology and blood eosinophilia. Vet Immun Immunopath.

[35] Chatamra K, Daniel PM, Kendall MD, Lam DK (1985). Atrophy of the thymus in rats rendered diabetic by strepozotocin. Horm Metab Res.

[36] Majno G, Palade GE, Schoefl GI (1961). Studies on inflammation. II. The site of action of histamine and serotonin along the vascular tree: a topographic study. J Biophys Biochem Cytol.

[37] Schoefl GI (1972). The migration of lymphocytes across the vascular endothelium in lymphoid tissue. A reexamination. J Exper Med.

[38] Savagner P, Imhof BA, Yamada KM, Thiery J-P (1986). Homing of hemopoietic precursor cells to the embryonic thymus: characterization of an invasive mechanism induced by chemotactic peptides. J Cell Biol.

[39] Dunon D, Imhof BA (1993). Mechanisms of thymus homing. Blood.

[40] Petri B, Bixel MG (2006). Molecular events during leukocyte diapedesis. FEBS J.

[41] Farr AG, De Bruyn PP (1975). The mode of lymphocyte migration through postcapillary venule endothelium in lymph node. Amer J Anat.

[42] Williams RM, Chanana AD, Cronkite EP, Waksman BH (1971). Antigenic markers on cells leaving calf thymus by way of the efferent lymph and venous blood. J Immunol.

[43] Chanana AD, Cronkite EP, Joel DD, Williams RM, Waksman BH (1971). Migration of thymic lymphocytes: immunofluorescence and 3HTdR labeling studies. Adv Exp Med Biol.

[44] Kotani M, Seiki K, Yamashita A, Horii I (1966). Lymphatic drainage of thymocytes to the circulation in the guinea pig. Blood.

[45] Ushiki T (1986). A scanning electron-microscopic study of the rat thymus with special reference to cell types and migration of lymphocytes into the general circulation. Cell Tissue Res.

[46] Miyasaka M, Pabst R, Dudler L, Cooper M, Yamaguchi K (1990). Characterization of lymphatic and venous emigrants from the thymus. Thymus.

[47] Azzali G, Orlandini G, Gatti R (1990). The migration of lymphocytes and polymorphonuclear leukocytes across the endothelial wall of the absorbing peripheral lymphatic vessel. J Submicrosc Cytol Pathol.

[48] Baluk P, Fuxe J, Hashizume H, Romano T, Lashnits E, Butz S, Vestweber D, Corada M, Molendini C, Dejana E, McDonald DM (2007). Functionally specialized junctions between endothelial cells of lymphatic vessels. J Exp Med.

[49] Lind EF, Prockop SE, Porritt HE, Petrie HT (2001). Mapping precursor movement through the postnatal thymus reveals specific microenvironments supporting defined stages of early lymphoid development. J Exp Med.

[50] Takahama Y (2006). Journey through the thymus: stromal guides for T-cell development and selection. Nat Rev Immunol.

[51] Wilson A, Capone M, MacDonald RM (1999). Unexpectedly late expression of intracellular CD3ε and TCR γδ proteins during adult thymus development. Int Immunol.

[52] Watanabe H, Seki S, Sugiura K, Hirokawa K, Utsuyama M, Takahashi-Iwanaga H, Iwanaga T, Ohteki T, Abo T (1992). Ontogeny and development of extrathymic T cells in liver. Immunology.

[53] Abo T (2001). Extrathymic pathways of T-cell differentiation and immunomodulation. Int Immunopharmacol.

[54] Narita S, Goldblum RM, Watson CS, Brooks EG, Estes DM, Curran EM, Midoro-Horiuti T (2007). Environmental estrogens induce mast cell degranulation and enhance IgE-mediated release of allergic mediators. Environ Health Perspect.

[55] Mobbs CV, Kannegieter LS, Finch CE (1985). Delayed anovulatory syndrome induced by estradiol in female C57BL/6 J mice: age-like neuroendocrine, but not ovarian impairments. Biol Reprod.

[56] Webb SJ, Geoghegan TE, Prough RA, Michael Miller KK (2006). The biological actions of dehydroepiandrosterone involves multiple receptors. Drug Metab Rev.

[57] Parker CR, Conway-Meyers BA (1998). The effects of dehydroepiandrosterone (DHEA) on the thymus, spleen, and adrenals of prepubertal and adult female rats. Endocr Res.

[58] Wilhelm M, King B, Silverman AJ, Silver R (2000). Gonadal steroids regulate the number and activational state of mast cells in the medial habenula. Endocrinology.

[59] Popova LA (1989). Effect of ovarian hormones on mast cells in the denervated uterus of rats. Fiziol Zh.

[60] Bartke A, Steele RE, Musto N, Caldwell BV (1973). Fluctuations in plasma testosterone levels in adult male rats and mice. Endocrinology.

[61] Chapman JC, Min S, Kunaporn S, Tung K, Shah S, Michael SD (2002). The administration of cortisone to female B6A mice during their immune adaptive period causes anovulation and the formation of ovarian cysts. Am J Reprod Immunol.

[62] Mitak M, Gojmerac T, Mandic B, Cvetnic Z (2001). Changes in serum concentrations of 17β-estradiol in female rats during estrous cycle after treatment with atrazine and zearalenone. Vet Med Czech.

[63] Lindberg MK, Weihua Z, Andersson N, Moverare S, Gao H, Vidal O, Erlandsson M, Windahl S, Andersson G, Lubahn DB, Carlsten H, Dahlman-Wright K, Gustafsson J-A, Ohlsson C (2002). Estrogen receptor specificity for the effects of estrogen in ovariectomized mice. J Endocrinol.

[64] Staples JE, Gasiewicz TA, Fiore NC, Lubahn DB, Korach KS, Silverstone AE (1999). Estrogen receptor α is necessary in thymic development and estradiol-induced thymic alterations. J Immunol.

[65] Anderson MS, Venanzi ES, Klein L, Chen Z, Berzins SP, Turley SJ, von Boehmer H, Bronson R, Dierich A, Benoist C, Mathis D (2002). Projection of an immunological self shadow within the thymus by the aire protein. Science.

[66] Pesic V, Radojevic K, Kosec D, Plecas-Sloarovic B, Perisic M, Leposavic G (2007). Peripubertal orchidectomy transitorily affects age-associated thymic involution in rats. Braz J Med Biol Res.

[67] Naquet P, Naspetti M, Boyd R (1999). Development, organization and function of the thymic medulla in normal, immunodeficient or autoimmune mice. Semin Immunol.

[68] Rotzschke O, Falk K, Deres K, Schild H, Norda M, Metzger J, Jung G, Rammwnsee H-G (1990). Isolation and analysis of naturally processed viral peptides as recognized by cytotoxic T cells. Nature.

[69] Girardi M (2006). Immunosurveillance and immunoregulation of γδ T cells. J Invest Dermatol.

[70] Takihara Y, Reimann J, Michalopoulos E, Ciccone E, Moretta L, Mak TW (1989). Diversity and structure of human T cell receptor delta chain genes in peripheral blood gamma/delta-bearing T lymphocytes. J Exp Med.

[71] Barakonyi A, Miko E, Varga P, Szekeres-Bartho J (2008). V-chain preference of gamma/delta T-cell receptors in peripheral blood during term labor. Am J Reprod Immunol.

[72] Cameron MJ, Kelvin DJ (2003). Cytokines and chemokines-their receptors and their genes: an overview. Adv Exp Med Biol.

[73] Kabelitz D, Peters C, Wesch D, Oberg HH (2013). Regulatory functions of γδ T cells. Int Immunopharmacol.

[74] Ebert EC, Roberts AI, Brolin RE, Raska K (1986). Examination of the low proliferative capacity of human jejunal intraepithelial lymphocytes. Clin Exp Immunol.

[75] Mincheva-Nilsson L, Hammarström S, Hammarström ML (1992). Human decidual leukocytes from early pregnancy contain high numbers of gamma delta + cells and show selective down-regulation of alloreactivity. J Immunol.

[76] Nagaeva O, Jonsson L, Mincheva-Nilsson L (2002). Dominant IL-10 and TGF-beta mRNA expression in gamma/delta T-cells of human early pregnancy decidua suggests immunoregulatory potential. Am J Reprod Immunol.

[77] Heyborne KD, Cranfill RL, Carding SR, Born WK, O'Brien RL (1992). Characterization of γδ T lymphocytes at the maternal-fetal interface. J Immunol.

[78] Fan D-X, Duan J, Li M-Q, Xu B, Li D-J, Jin L-P (2011). The decidual gamma-delta T cells up-regulate the biological functions of trophoblasts via IL-10 secretion in early human pregnancy. Clin Immunol.

[79] Groux H (2001). An overview of regulatory T cells. Microbes Infect.

[80] Ballas ZK, Rasmussen W (1990). NK1.1+ thymocytes. Adult murine CD4-, CD8- thymocytes contain an NK1.1+, CD3+, CD5hi, CD44hi, TCR-V beta 8+ subset. J Immunol.

[81] Zlotnik A, Godfrey DI, Fischer M, Suda T (1992). Cytokine production by mature and immature CD4-CD8- T cells. Alpha beta-T cell receptor + CD4-CD8- T cells produce IL-4. J Immunol.

[82] Cowley SC, Hamilton E, Frelinger JA, Su J, Forman J, Elkins KL (2005). CD4-CD8- T cells control intracellular bacterial infections both in vitro and in vivo. J Exp Med.

[83] Geirsson A, Paliwal I, Lynch RJ, Bothwell AL, Hammond GL (2003). Class II transactivator promoter activity is suppressed through regulation by a trophoblast noncoding RNA. Transplantation.

[84] Barakonyi A, Kovacs KT, Miko E, Szereday L, Varga P, Szekeres-Bartho J (2002). Recognition of nonclassical HLA class I antigens by gamma delta T cells during pregnancy. J Immunol.

[85] Park D-W, Yang K-M (2011). Hormonal regulation of uterine chemokines and immune cells. Clin Exp Reprod Med.

[86] Wu X, Jin LP, Yuan MM, Zhu Y, Wang MY, Li DJ (2005). Human first-trimester trophoblast cells recruit CD56^bright^CD16^—^ NK cells into decidua by way of expressing and secreting of CXCL12/stromal cell-derived factor 1. J Immunol.

[87] Hanna J, Wald O, Goldman-Wohl D, Prus D, Markel G, Gazit R, Katz G, Haimov-Kochman R, Fujii N, Yagel S, Peled A, Mandelboim O (2003). CXCL12 expression by invasive trophoblasts induces the specific migration of CD16^—^ human natural killer cells. Blood.

[88] Huang Y, Zhu XY, Du MR, Li DJ (2008). Human trophoblasts recruited T lymphocytes and monocytes into deciduas by secretion of CXCL16 and interaction with CXCR6 in the first-trimester pregnancy. J Immunol.

[89] Dimova T, Nagaeva O, Stenqvist A-C, Hedlund M, Kjellberg L, Strand M, Dehlin E, Mincheva-Nilsson L (2011). Maternal foxp3 expressing CD4+ CD25+ and CD4+ CD25^—^regulatory T-cell populations are enriched in human early normal pregnancy decidua: a phenotypic study of paired decidual and peripheral blood samples. Am J Reprod Immunol.

[90] Blois SM, Kammerer U, Alba Soto C, Tometten MC, Shaikly V, Barrientos G, Jurd R, Rukavina D, Thomson AW, Klapp BF, Fernández N, Arck PC (2007). Dendritic cells: key to fetal tolerance?. Biol Reprod.

[91] Maroni ES, de Sousa MA (1973). The lymphoid organs during pregnancy in the mouse. A comparison between syngeneic and allogeneic mating. Clin Exp Immunol.

[92] Gray H (1985). Anatomy of the human body.

[93] Koukourakis MI, Giatromanolaki A, Sivridis E, Simopoulos C, Gatter KC, Harris AL, Jackson DG (2005). LYVE-1 immunohistochemical assessment of lymphangiogenesis in endometrial and lung cancer. J Clin Pathol.

[94] Red-Horse K (2008). Lymphatic vessel dynamics in the uterine wall. Placenta.

[95] Arck PC, Ferrick DA, Steele-Norwood D, Egan PJ, Croitoru K, Carding SR, Dietl J, Clark DA (1999). Murine T cell determination of pregnancy outcome. Cell Immunol.

[96] Arck PC, Ferrick DA, Steele-Norwood D, Croitoru K, Clark DA (1997). Murine T cell determination of pregnancy outcome: I. effects of strain, alpha/beta T cell receptor, gamma/delta T cell receptor, and gamma/delta T cell subsets. Am J Reprod Immunol.

[97] Goncalves-Sousa N, Ribot JC, de Barros A, Correia DV, Caramalho I, Silva-Santos B (2010). Inhibition of murine gammadelta lymphocyte expansion and effector function by regulatory alphabeta T cells is cell-contact-dependent and sensitive to GITR modulation. Eur J Immunol.

[98] Mahan CS, Thomas JJ, Boom WH, Rojas RE (2009). CD4^+^ CD25^high^ Foxp3^+^ regulatory T cells downregulate human Vδ2^+^ T-lymphocyte function triggered by anti-CD3 or phosphoantigen. Immunology.

[99] Kallikourdis M, Betz AG (2007). Periodic Accumulation of Regulatory T Cells in the Uterus: Preparation for the Implantation of a Semi-Allogeneic Fetus?. PLoS One.

[100] Aluvihare VR, Kallikourdis M, Betz AG (2004). Regulatory T cells mediate maternal tolerance to the fetus. Nat Immunol.

[101] Somerset DA, Zheng Y, Kilby MD, Sansom DM, Drayson MT (2004). Normal human pregnancy is associated with an elevation in the immune suppressive CD25^+^ CD4^+^ regulatory T-cell subset. Immunology.

[102] Barakonyi A, Polgar B, Szekeres-Bartho J (1999). The role of gamma/delta T-cell receptor-positive cells in pregnancy: part II. Am J Reprod Immunol.

[103] Szekeres-Bartho J, Barakonyi A, Polgar B, Par G, Faust Z, Palkovics T, Szereday L (1999). The role of γδ T cells in progesterone-mediated immunomodulation during pregnancy: a review. Am J Reprod Immunol.

[104] Hara T, Mizuno Y, Takaki K, Akeda H, Aoki T, Nagata M, Ueda K, Matsuzaki G, Yoshikai Y (1992). Predominant activation and expansion of Vγ9-bearing γδ T cells in vivo as well as in vitro in Salmonella infection. J Clin Invest.

[105] Munk ME, Gatrill AJ, Kaufmann SH (1990). Target cell lysis and IL-2 secretion by γδ T lymphocytes after activation with bacteria. J Immunol.

[106] Du MR, Guo PF, Piao HL, Wang SC, Sun C, Jin LP, Tao Y, Li YH, Zhang D, Zhu R, Fu Q, Li DJ (2014). Embryonic trophoblasts induce decidual regulatory T cell differentiation and maternal–fetal tolerance through thymic stromal lymphopoietin instructing dendritic cells. J Immunol.

[107] Sasaki Y, Sakai M, Miyazaki S, Higuma S, Shiozaki A, Saito S (2004). Decidual and peripheral blood CD4+ CD25+ regulatory T cells in early pregnancy subjects and spontaneous abortion cases. Mol Hum Reprod.

[108] Winger EE, Reed JL (2011). Low circulating CD4(+) CD25(+) Foxp3(+) T regulatory cell levels predict miscarriage risk in newly pregnant women with a history of failure. Am J Reprod Immunol.

[109] Zygmunt M, Herr F, Münstedt K, Lang U, Liang OD (2003). Angiogenesis and vasculogenesis in pregnancy. Eur J Obstet Gynecol Reprod Biol.

[110] Olivares EG, Munoz R, Tejerizo G, Montes MJ, Gomez-Molina F, Abadia-Molina AC (2002). Decidual lymphocytes of human spontaneous abortions induce apoptosis but not necrosis in JEG-3 extravillous trophoblast cells. Biol Reprod.

[111] King A, Jokhi PP, Burrows TD, Gardner L, Sharkey AM, Loke YW (1996). Functions of human decidual NK cells. Am J Reprod Immunol.

[112] Keskin DB, Allan DS, Rybalov B, Andzelm MM, Stern JN, Kopcow HD, Koopman LA, Strominger JL (2007). TGF beta promotes conversion of CD16+ peripheral blood NK cells into CD16^—^ NK cells with similarities to decidual NK cells. Proc Natl Acad Sci U S A.

[113] Kopcow HD, Allan DS, Chen X, Rybalov B, Andzelm MM, Ge B, Strominger JL (2005). Human decidual NK cells form immature activating synapses and are not cytotoxic. Proc Natl Acad Sci U S A.

[114] Sharkey DJ, Macpherson AM, Tremellen KP, Mottershead DG, Gilchrist RB, Robertson SA (2012). TGF-β mediates proinflammatory seminal fluid signaling in human cervical epithelial cells. J Immunol.

[115] Nocera M, Chu TM (1995). Characterization of latent transforming growth factor-beta from human seminal plasma. Am J Reprod Immunol.

[116] Tremellen KP, Seamark RF, Robertson SA (1998). Seminal Transforming Growth Factor 1, Stimulates Granulocyte-Macrophage Colony-Stimulating Factor Production and Inflammatory Cell Recruitment in the Murine Uterus. Biol Reprod.

[117] Shooner C, Caron PL, Fréchette-Frigon G, Leblanc V, Déry MC, Asselin E (2005). TGF-beta expression during rat pregnancy and activity on decidual cell survival. Reprod Biol Endocrin.

[118] Aoki K, Kajiura S, Matsumoto Y, Oqasawara M, Okada S, Yaqami Y, Gleicher N (1995). Preconceptional natural-killer-cell activity as a predictor of miscarriage. Lancet.

[119] Higuchi K, Aoki K, Kimbara T, Hosoi N, Yamamoto T, Okada H (1995). Suppression of natural killer cell activity by monocytes following immunotherapy for recurrent spontaneous aborters. Am J Reprod Immunol.

[120] Lachapelle MH, Miron P, Hemmings R, Roy DC (1996). Endometrial T, B, and NK cells in patients with recurrent spontaneous abortion. Altered profile and pregnancy outcome. J Immunol.

[121] Clifford K, Flanagan AM, Regan L (1999). Endometrial CD56+ natural killer cells in women with recurrent miscarriage: a histomorphometric study. Hum Reprod.

[122] Emmer PM, Nelen WL, Steegers EA, Hendriks JC, Veerhoek M, Joosten I (2000). Peripheral natural killer cytotoxicity and CD56+ CD16+ cells increase during early pregnancy in women with a history of recurrent spontaneous abortion. Hum Reprod.

[123] Park DW, Lee HJ, Park CW, Hong SR, Kwak-Kim J, Yang KM (2010). Peripheral blood NK cells reflect changes in decidual NK cells in women with recurrent miscarriages. Am J Reprod Immunol.

[124] Junovich G, Azpiroz A, Incera E, Ferrer C, Pasqualini A, Gutierrez G (2013). Endometrial CD16(+) and CD16(−) NK cell count in fertility and unexplained infertility. Am J Reprod Immunol.

[125] Seshadri S, Sunkara SK (2014). Natural killer cells in female infertility and recurrent miscarriage: a systematic review and meta-analysis. Hum Reprod Update.

[126] Abbassi-Ghanavati M, Greer LG, Cunningham FG (2009). Pregnancy and laboratory studies: a reference table for clinicians. Obstet Gynecol.

[127] Heyborne K, Fu YX, Nelson A, Farr A, O'Brien R, Born W (1994). Recognition of trophoblasts by gamma delta T cells. J Immunol.

[128] Ashkar AA, Di Santo JP, Croy BA (2000). Interferon gamma contributes to initiation of uterine vascular modification, decidual integrity, and uterine natural killer cell maturation during normal murine pregnancy. J Exp Med.

[129] Murphy SP, Tayade C, Ashkar AA, Hatta K, Zhang J, Croy BA (2009). Interferon gamma in successful pregnancies. Biol Reprod.

[130] Folkman J, Klagsbrun M (1987). Angiogenic factors. Science.

[131] Seo N, Tokura Y, Takigawa M, Egawa K (1999). Depletion of IL-10 and TGF-β-producing γδ T cells by administering a daunomycin-conjugated specific monoclonal antibody in early tumor lesions augments the activity of CTLs and NK cells. J Immunol.

[132] Abo T, Sugawara S, Seki S, Fujii M, Rikiishi H, Takeda K, Kumagai K (1990). Induction of human TCR gamma delta + and TCR gamma delta-CD2 + CD3- double negative lymphocytes by bacterial stimulation. Int Immunol.

[133] Abo T, Kusumi A, Seki S, Ohteki T, Sugiura K, Masuda T, Rikiishi H, Iiai T, Kumagai K (1992). Activation of extrathymic T cells in the liver and reciprocal inactivation of intrathymic T cells by bacterial stimulation. Cell Immunol.

[134] Seki S, Abo T, Sugiura K, Ohteki T, Kobata T, Yagita H, Okumura K, Rikiishi H, Masuda T, Kumagai K (1991). Reciprocal T cell responses in the liver and thymus of mice injected with syngeneic tumor cells. Cell Immunol.

